# Alcohol Expectancies Mediate and Moderate the Associations between Big Five Personality Traits and Adolescent Alcohol Consumption and Alcohol-Related Problems

**DOI:** 10.3389/fpsyg.2015.01838

**Published:** 2015-11-26

**Authors:** Manuel I. Ibáñez, Laura Camacho, Laura Mezquita, Helena Villa, Jorge Moya-Higueras, Generós Ortet

**Affiliations:** ^1^Department of Basic and Clinical Psychology and Psychobiology, Universitat Jaume ICastellón de la Plana, Spain; ^2^Centre for Biomedical Research Network on Mental Health (CIBERSAM), Instituto de Salud Carlos IIIMadrid, Spain; ^3^Department of Pedagogy and Psychology, Universitat de LleidaLleida, Spain

**Keywords:** personality, five-factor model, expectancies, adolescents, mediation, moderation, alcohol

## Abstract

Personality and expectancies are relevant psychological factors for the development of adolescent alcohol use and misuse. The present study examined their direct, mediated and moderated effects on different drinking behaviors in adolescence. Personality domains of the five-factor model, positive and negative alcohol expectancies (AEs), alcohol use during the week and the weekend, and alcohol-related problems were assessed in a sample of 361 adolescents. Different personality dimensions were directly associated with specific alcohol outcomes: Extraversion, low Conscientiousness and low Openness were associated with weekend alcohol use; low Agreeableness was related to weekday use; whereas low Agreeableness, low Conscientiousness and Extraversion were associated with alcohol-related problems. In addition, positive AEs mediated the relationship between Extraversion and alcohol use, whereas both positive and negative expectancies mediated the association between Neuroticism and alcohol consumption and alcohol-related problems. Finally, both types of expectancies interacted with Extraversion to predict alcohol problems. Our results highlight the importance of examining the complex interplay of comprehensive personality models and AEs to gain a better understanding of the development of different alcohol use and misuse patterns in adolescence.

## Introduction

Alcohol use is typically initiated and extended in adolescence (Marshall, 2014). The mean age of drinking initiation in Spain is 13.6 years, whereas 84% of 16-year-olds have drunk over the last year (National Plan of Drugs, 2013). Adolescent drinking should be a special matter of concern because it is the main risk factor that contributes to disability-adjusted life-years (DALYs) worldwide (Gore et al., 2011), and is associated with alcoholism and other negative outcomes in adulthood (McCambridge et al., 2011). Consequently, defining those factors that lead adolescents to use alcohol is essential to develop more effective prevention programs. Two of the most relevant psychological factors are alcohol expectancies (AEs) and basic personality traits.

Nowadays, the most widely used and integrative model of personality traits is the five-factor model (FFM; John et al., 2008), which encompasses five personality dimensions: Extraversion, Neuroticism, Agreeableness, Conscientiousness, and Openness to Experience (McCrae and Costa, 2008). Accordingly, the role of the FFM on adult drinking behavior has been extensively examined and several meta-analyses have been conducted. These studies have established the relevance of low Conscientiousness on the use and misuse of alcohol, and of low Agreeableness and high Neuroticism on alcohol-related problems and pathological alcohol use (Malouff et al., 2007; Kotov et al., 2010).

The importance of personality traits, and impulsivity/disinhibition characteristics in particular, has also been widely documented for adolescent drinking (Stautz and Cooper, 2013), although FFM domains have been seldom explored at these ages. The scarce data available suggest that, unlike what is found in adulthood, Extraversion is the most relevant personality characteristic in adolescent alcohol use, whereas the relevance of other FFM domains in different adolescent drinking outcomes is still unclear (Merenäkk et al., 2003; van der Zwaluw et al., 2010; Pilatti et al., 2013). This is a large gap in alcohol research because the FFM constitutes a bridge to integrate models of youth and adult personality traits (Caspi et al., 2005); thus the assessment of FFM domains at earlier ages could allow the direct comparison of the role of personality traits on drinking behavior across different developmental stages from childhood to adulthood.

From a biodispositional perspective, however, personality traits are considered a distal, non-specific variables that would influence alcohol use through more proximal and specific variables, such as expectancies (Ibáñez et al., 2008). AEs are cognitive structures that refer to the beliefs about the positive (i.e., “If I drink alcohol, I will be friendlier”) and negative (i.e., “If I drink alcohol, I will have a hangover”) consequences that this substance produces at emotional, motivational, and behavioral levels. Accordingly, positive AEs have been strongly associated with adolescent drinking behaviors, whereas negative AEs have shown a slightly protective role for alcohol use and abuse in adolescence (Brown et al., 1987; Leigh and Stacy, 2004; Corbin et al., 2011).

Although AEs develop from direct and indirect experiences with alcohol, personality characteristics may shape these experiences. Thus the Acquired Preparedness Model considers that individuals are differentially prepared to acquire certain learning experiences according to their personalities (Smith and Anderson, 2001; Settles et al., 2010). It has been particularly hypothesized that Reward Sensitivity characteristics (sensitivity to rewarding stimuli and strength of motivation to obtain them; closely linked to Extraversion) would be associated with reward-related learning, which may lead to develop positive expectancies of alcohol outcomes which, in turn, would promote alcohol use (Gullo et al., 2010). Accordingly, studies conducted in adulthood have confirmed this mediational role for Positive Expectancies in the association of Extraversion and alcohol consumption (Read and O’Connor, 2006; Gullo et al., 2010; Mezquita et al., 2015). Other personality domains related to low Conscientiousness and low Agreeableness have also been seen as relevant for adult substance use, but not through the expression of more positive AEs (Finn et al., 2000; Gullo et al., 2010; Mezquita et al., 2015). Lastly, Neuroticism seems relevant for negative AEs, but also for positive ones. Specifically, Neuroticism has been associated with alcohol problems through negative AEs, and also with alcohol use through positive AEs (Read and O’Connor, 2006; Mezquita et al., 2015).

The role of FFM in the development of AEs in adolescence, a key stage for the development of AEs and drinking behavior (Leigh and Stacy, 2004), remains almost unexplored. To our knowledge, only one study has examined the interplay between two FFM dimensions, Extraversion and Conscientiousness, positive and negative AEs and adolescent drinking frequency, which found that positive AEs mediated the association between Extraversion and frequency of alcohol use, which parallels adult findings (Pilatti et al., 2012).

Finally, and as far as we know, only one study has explored the interactive role of expectancies and FFM personality domains in adulthood. Mezquita et al. (2015) found that adults with more positive AEs showed a stronger association between weekend drinking and low Conscientiousness. For alcohol problems, this study reported an interaction effect between positive expectancies and Neuroticism, and between both positive and negative expectancies and low Agreeableness. Whether similar synergistic effects are also present in adolescence is yet to be explored.

Thus the main aim of this study was to systematically examine the role of personality traits and AEs in adolescent alcohol use and misuse. To this end, we used the FFM, included both positive and negative AEs, and assessed relevant alcohol-related behaviors such as alcohol problems and weekday and weekend alcohol use (Ibáñez et al., 2010; Studer et al., 2014), and explored mediation and moderation effects. The main hypotheses were that Extraversion, low Conscientiousness and low Agreeableness predict alcohol consumption. As alcohol use during the week represents a more antinormative behavior than weekend alcohol use (Mezquita et al., 2014), we expected Extraversion and low Conscientiousness to associate with weekend alcohol use, and the domain more related to antisocial behavior, low Agreeableness, to relate to drinking during the week. We also expected low Conscientiousness, low Agreeableness and Neuroticism to be associated with alcohol problems. We expected positive AEs to be associated with drinking behavior, and negative AEs to be less relevant and negatively related to alcohol use. For mediation effects, we hypothesized that the association between Extraversion and alcohol use is mediated by positive AEs. In addition, Neuroticism would be associated with both positive and negative AEs. Finally for moderation effects, and based on adult findings within the FFM framework, we expected positive expectancies to interact with low Conscientiousness and Neuroticism to predict weekend alcohol use and alcohol problems, respectively; and both positive and negative expectancies to interact with low Agreeableness to predict alcohol problems.

## Materials and Methods

### Participants and Procedure

Seven high schools from rural and urban areas participated in this study. Research assistants asked students to answer the questionnaires in class during two different sessions, and helped them whenever necessary. Of the 428 students invited to participate, 84% returned a signed parental written consent and completed the questionnaires in both sessions. The final sample was composed of 361 adolescents aged 14–16 years (149 males and 212 females; mean age = 15.16, *SD* = 0.60). Most were born in Spain (88.3%). The percentage of foreigners in this sample is in accordance with their distribution in high schools in Spain (National Plan of Drugs, 2013).

### Ethics

This study was carried out in accordance with the recom mendations of the ethical committee from the Universitat Jaume I. Parents or legal tutors of the participants gave written informed consent in accordance with the Declaration of Helsinki.

### Measures

#### Personality Traits

We used the Spanish adaptation of the NEO-PI-R for adolescents, the JS NEO (Ortet et al., 2012). This self-report questionnaire includes 150 items that are answered on a 5-point Likert scale, and ranges from *strongly disagree* to *strongly agree*. It assesses the five personality factors or domains: Neuroticism, Extraversion, Openness to Experience, Agreeableness and Conscientiousness. The internal consistency indices ranged from α = 0.78 for Agreeableness to α = 0.90 for Conscientiousness (see the Supplementary Table S1 for all the scales).

#### Alcohol Expectancies

We used the Spanish adaptation in adolescents of the Expectancy Questionnaire (EQ; Camacho et al., 2013). The scale consists of 34 items and takes a 6-point Likert format that measures positive and negative AEs. Positive AEs (19 items) comprised expectancies about social facilitation, positive affect potentiation, sexual disinhibition and tension reduction; Negative AEs (15 items) included expectancies about antisocial effects of alcohol, negative emotional states, as well as undesirable physical and cognitive effects. Items are short phrases prefaced by *When I drink alcohol*… Respondents had to indicate the likelihood of the indicated consequences happening to them when they drink. Non-drinkers were asked to answer according to what they thought would have happened if they had drunk. In this study, the internal consistency indices for positive AEs was α = 0.95 and was α = 0.92 for negative AEs.

#### Alcohol Use and Misuse

We used the AIS-UJI, a self-report scale in which participants indicated the quantity of glasses of beer, wine, liquors, and mix drinks they drank during the week (from Monday to Thursday) and at the weekend (from Friday to Sunday). The informed drinks were transformed into Standard Drink Units (1 SDU = 10 g of alcohol; see Mezquita et al., 2015).

Finally, we used the Alcohol Use Disorders Identification Test (AUDIT; Babor et al., 2001). AUDIT includes 10 items on a 3- and 5-point Likert scale, which are grouped into three “alcohol consumption,” “alcohol dependence” and “harmful alcohol use” subscales. We used the last two scales (seven items) to assess alcohol-related problems, which presented an internal consistency of α = 0.72.

### Data Analyses

We conducted the descriptive analyses, *t*-tests, Cronbach’s alphas and correlations with the SPSS statistic package, version 21. The same software was used to carry out the regression analyses in order to explore the interaction between: (a) personality and drinking status (non-drinkers vs. drinkers) as predictors of AEs; and (b) personality and AEs as predictors of alcohol outcomes. We introduced the variables as follows: age, sex in a first step; personality domains in a second step; (a) drinking status, or (b) AEs (positive and negative separately) in a third step; and the product term between each personality scale and (a) drinking status or (b) positive or negative AEs in a fourth step. Personality and AEs scales were centered (Aiken and West, 1991).

In order to explore the direct and indirect relationships of personality and AEs to alcohol use and alcohol problems, we also performed path analyses with the EQS software, version 6.1. Robust methods were used given the non-normality in the data. The model’s goodness-of-fit was evaluated using the following fit indices: Satorra-Bentler chi-squared (_S-B_χ^2^), normed chi-squared (_S-B_χ^2^/d.f.), the comparative fit index (CFI), the incremental fit index (IFI), the non-normal fit index (NNFI), and the root mean square error of approximation (RMSEA). For a model to show a good fit, _S-B_χ^2^ had to be non-significant, the normed _S-B_χ^2^ had to be between 1 and 2, CFI, IFI, and NNFI had to be.95 or higher, and RMSEA had to be 0.05 or lower (Byrne, 2006). Having obtained the final model in the total sample, this model was tested in the drinkers subgroup in order to compare paths similarities and differences between drinkers and the whole sample.

## Results

The pattern of alcohol use in our sample was similar to what is usually found in Spanish adolescents (Llorens et al., 2011; National Plan of Drugs, 2013). Specifically, 56% of the sample informed that they drunk alcohol (202 drinkers), with a range of 1–50 SDUs, and a mean of 9.98 SDUs (*SD* = 8.77). Of the remaining 44% (159 non-drinkers), 45% informed that never consumed alcohol (72 abstainers). In addition, the percentage of drinkers was higher among boys than among girls (59% vs. 53%, respectively). Thus, we explored the mean differences according to gender and drinking status (non-drinkers vs. drinkers). Females obtained higher mean scores than males in Neuroticism, Openness, and Agreeableness, whereas males obtained higher mean scores than females in quantity of drinking. We also found that drinkers were more extraverted, less conscientious, and presented a much larger number of positive AEs than non-drinkers; that is, they presented mean differences in the usual variables that associate with alcohol use (see Supplementary Table S1).

Despite these mean differences, the regression analysis did not show significant interactions between drinking status and the FFM domains for predicting AEs (see the Supplementary Table S2). These data indicate that the pattern of associations between personality and AEs did not differ between drinkers and non-drinkers.

**FIGURE 1:**
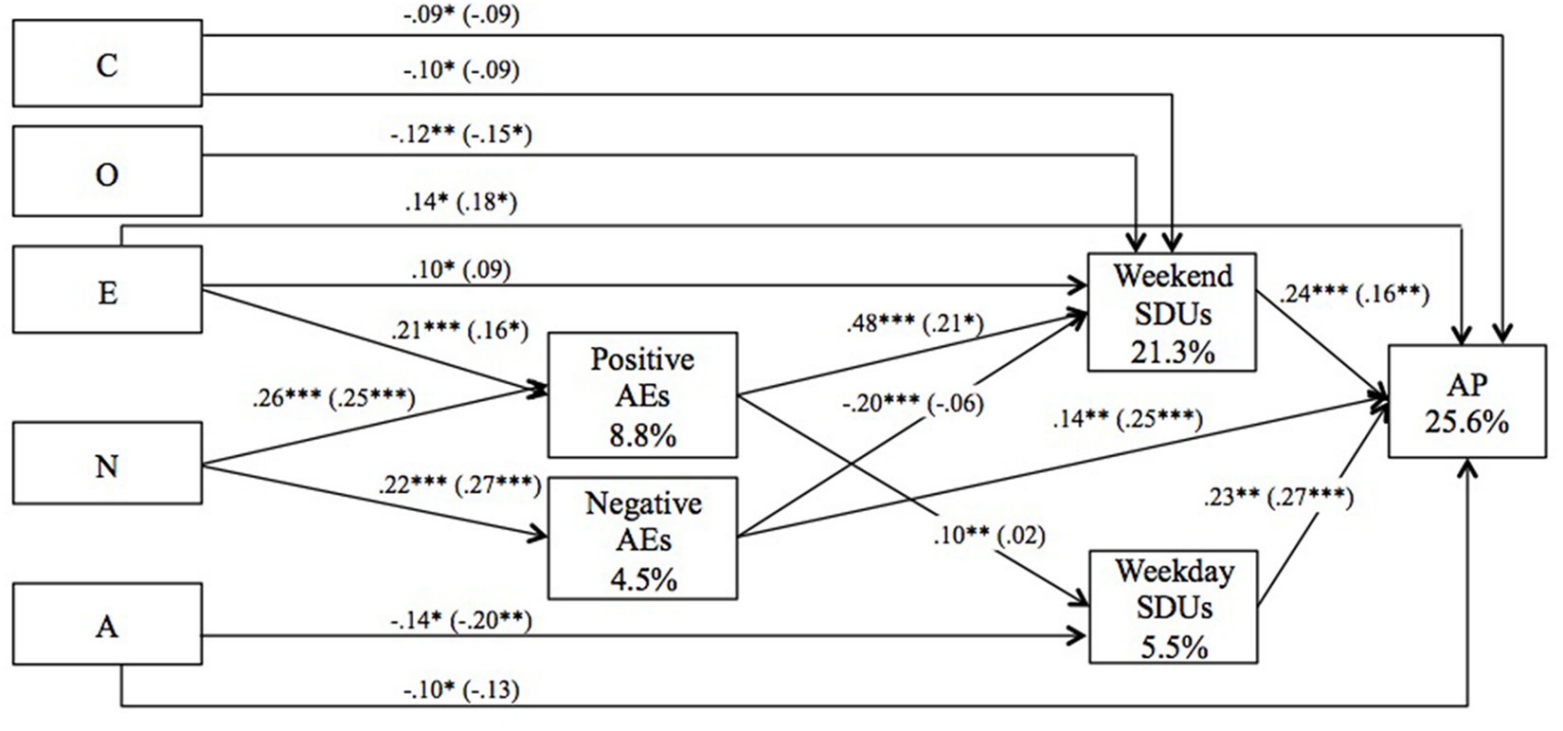
**Final path model.** Standardized β coefficients for the total sample *(N* = 361) and for the drinkers group *(N* = 202; in parentheses) are represented at ^∗^*p* < 0.05; ^∗∗^*p* < 0.01; ^∗∗∗^*p* < 0.001. Boxes show the percentages of explained variance (*R*^2^) for the total sample. The correlations among personality dimensions, expectancies and SDUs are not included in the figure because of space restrictions. Gender was covaried with all the variables to control its effect. N, Neuroticism; E, Extraversion; O, Openness to Experience; A, Agreeableness; C, Conscientiousness; SDUs, Standard Drink Units; AEs, Alcohol Expectancies; AP, Alcohol-related Problems.

We tested the hypothesized model on the total sample, and it showed adequate fit indices [_S-B_χ^2^(17, *N* = 361) = 38.56; *p* = 0.002; _S-B_χ^2^/d.f. = 2.27; CFI = 0.961; NNFI = 0.873; IFI = 0.963; RMSEA = 0.059]. However, after removing the non-significant paths as suggested by the Wald test (Neuroticism, positive AEs on alcohol problems; negative AEs on Weekday SDUs) and adding new paths as suggested by the LM Test (Extraversion, Openness on Weekend SDUs; Extraversion on alcohol problems), the fit indices of the final model were excellent [_S-B_χ^2^(17, *N* = 361) = 17.35; *p* = 0.43; _S-B_χ^2^/d.f. = 1.02; CFI = 0.999; NNFI = 0.998; IFI = 0.999; RMSEA = 0.008] (see **Figure 1**). **Table 1** presents the indirect and total effects. In addition, the final model was tested in the drinkers group. The fit indices were excellent [_S-B_χ^2^(17, *N* = 202) = 18.42; *p* = 0.36; _S-B_χ^2^/d.f. = 1.08; CFI = 0.995; NNFI = 0.983; IFI = 0.995; RMSEA = 0.020] (see **Figure 1**). Most of the β coefficient magnitudes were similar to those with the total sample, although some became non-significant because of sample size reduction. Only the β coefficients magnitudes between expectancies and alcohol outcomes notably modified when non-drinkers were excluded, which probably indicated a range restriction issue.

**Table 1:** Indirect and total effects of the final path analysis.

Path	St. β	Path	St. β
**Indirect effects**			
N → Weekday SDUs	0.03*	O → AP	-0.03*
E → Weekday SDUs	0.02*	A → AP	-0.03*
N → Weekend SDUs	0.08**	C → AP	-0.02*
E → Weekend SDUs	0.10***	Positive AEs → AP	0.14***
N → AP	0.06**	Negative AEs → AP	-0.05**
E → AP	0.05**		
**Total effects**			
N → Positive AEs	0.26***	Positive AEs → Weekend SDUs	0.48***
E → Positive AEs	0.21***	Negative AEs → Weekend SDUs	-0.20***
N → Negative AEs	0.22***	N → AP	0.06***
N → Weekday SDUs	0.03*	E → AP	0.19***
E → Weekday SDUs	0.02*	O → AP	-0.03*
A → Weekday SDUs	-0.14*	A → AP	-0.14**
AEs → Weekday SDUs	0.10**	C → AP	-0.12*
N → Weekend SDUs	0.08**	Positive AEs → AP	0.14***
E → Weekend SDUs	0.20***	Negative AEs → AP	0.09
O → Weekend SDUs	-0.12**	Weekday SDUs → AP	0.23**
C → Weekend SDUs	-0.10*	Weekend SDUs → AP	0.24***

*N, Neuroticism; E, Extraversion; O, Openness to Experience; A, Agreeableness; C, Conscientiousness; SDUs, Standard Drink Units; AEs, Alcohol Expectancies; AP, Alcohol-related Problems. ^∗^*p* < 0.05; ^∗∗^*p* < 0.01; ^∗∗∗^*p* < 0.001.*

**FIGURE 2:**
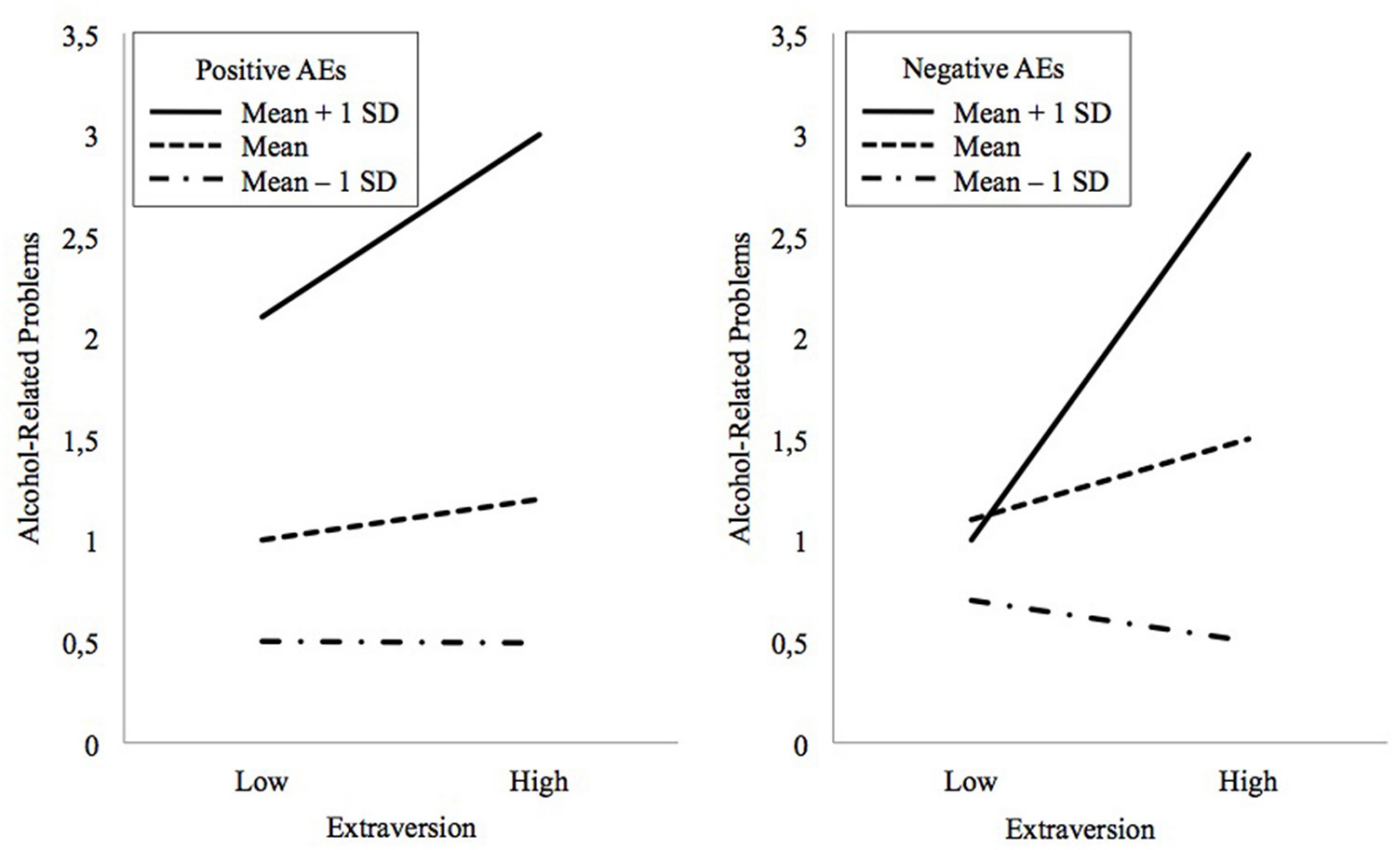
**Effects of Extraversion on Alcohol-Related Problems moderated by positive **(left)** and negative **(right)** alcohol expectancies (AEs)**.

With respect to moderation effects, we conducted a series of hierarchical regressions on weekday SDUs, weekend SDUs and alcohol problems. Two interactions, Extraversion × positive AEs (β = 0.14, *p* < 0.01) and Extraversion × negative AEs (β = 0.21, *p* < 0.001), predicted alcohol problems (see the Supplementary Table S3). These interactions were maintained even when controlled by the total amount of alcohol consumed, by age × AEs, and by gender × AEs. **Figure 2** indicates the direction of the moderation effects.

As we found that Positive Expectancies mediated the association between Extraversion and alcohol outcomes, and that Positive Expectancies moderated the relationship between Extraversion and alcohol problems, a moderated mediation effect was also tested (see Muller et al., 2005). In order to test a moderated mediation effect in the final model (**Figure 1**), we explored if the mediation effect in the relationship between Extraversion and alcohol-related problems exists at different levels of Extraversion. Thus, a multi-group analysis between participants that scored high and low on extraversion (above and below the mean) was performed. The multi-group model showed adequate fit indices [_S-B_χ^2^(34, *N* = 361) = 39.75; *p* = 0.229; _S-B_χ^2^/d.f. = 1.17; CFI = 0.989; NNFI = 0.964; IFI = 0.990; RMSEA = 0.031]. When we constrained the paths between positive expectancies and drinking during the week and at the weekend, there was not a significant decrement in fit [_S-B_χ^2^diff (2) = 1.63, *p* = 0.44]. This result suggests that the mediation of positive expectancies in the relationship between Extraversion and alcohol-related problems is not moderated by Extraversion. Splitting the sample into subgroups that represent different values of the moderator variable has several drawbacks (see Edwards and Lambert, 2007), so we conducted an additional moderated mediation analysis as suggested by Preacher et al. (2007) using the PROCESS macros for SPSS provided by Hayes (2015; http://www.processmacro.org). The analysis utilized the conditional indirect effect model posited by Model 74 of PROCESS, in our case with Extraversion as *x*, Positive Expectancies as *m*, and Alcohol Problems as *y*. The results of this analysis were in accordance with multi-group analysis, and indicate that there was not a moderated mediation effect (index of moderated mediation = 0.0492; *SE* = 0.0360; 95% bootstrap confidence interval from -0.0002 to 0.1408; see Hayes, 2015).

## Discussion

Personality traits and AEs are two of the most relevant and studied psychological factors for alcohol use. Nevertheless, very little is known about the interplay of FFM dimensions and positive and negative AEs in adolescent alcohol use and misuse. This is precisely what the present study explored.

Regarding personality, all the FFM domains were associated with adolescent drinking, although distinct dimensions influenced specific patterns of alcohol use. Extraversion (specifically gregariousness and excitement seeking facets; see Supplementary Table S4), and low Conscientiousness (mainly low deliberation), predicted greater alcohol consumption at the weekend, whereas low Agreeableness (particularly low compliance and low modesty) was associated with weekday SDUs, as hypothesized. A similar distinctive association has been described in young adults (Mezquita et al., 2015), which supports the importance of distinguishing between weekday and weekend alcohol use (Studer et al., 2014). One unexpected finding was the slight association of low Openness with weekend SDUs. As weekend alcohol consumption has become a normative activity among Spanish adolescents, it is possible that high Openness youngsters may be more involved in alternative recreational activities in which alcohol is less present (e.g., going to the cinema, playing video games, etc.) than low Openness adolescents. Finally, high Extraversion and Neuroticism, and low Agreeableness, Conscientiousness and Openness have been associated with alcohol problems, either directly or indirectly through alcohol use. These results indicate that the development of alcohol problems in adolescents is associated with high alcohol use, along with a more extraverted, impulsive, conventional, and neurotic personality profile.

Regarding expectancies, positive AEs related strongly to all the drinking outcomes. The role of negative AEs is less clear. Even though the path analysis showed a *negative* parameter at weekend SDUs (see **Figure 1**), the correlations were non-significant (see Supplementary Table S5). We believe that the apparently protective role of negative AEs indeed reflects a statistical suppression effect: an initial predictor (positive AEs) benefits from the entry of the new predictor, which appears to have no real predictive power (*negative AEs*) and is manifested in the negative beta weight for the new predictor (Paulhus et al., 2004). In any case, our results confirm the notion that positive AEs are much better predictors of adolescent alcohol use than negative AEs (Leigh and Stacy, 2004). Although negative AEs seem irrelevant for alcohol use, they were positively and directly associated with alcohol problems, which falls in line with previous studies conducted in adults (Read and O’Connor, 2006; Spillane et al., 2012). As it is unlikely that more expectancies of negative alcohol outcomes will lead to more problematic alcohol use, it has been suggested that negative AEs are more likely to be the result of problematic alcohol use rather than being an antecedent for it (Spillane et al., 2012).

Hypotheses about the mediational role of positive AEs were confirmed. The results provided evidence for partial mediation of the association between Extraversion and alcohol use, but showed no evidence of mediation by expectancies for the associations between Conscientiousness and Agreeableness and alcohol use. This suggests that different personality characteristics would lead to distinct alcohol use patterns through different etiological pathways. Thus Extraversion-related characteristics (mainly the excitement seeking facet, see Supplementary Table S4) would be involved on a Positive Affect regulation pathway, on which positive expectancies and motives would lead to more recreational alcohol use (Sher et al., 2005; Ibáñez et al., 2008; Mezquita et al., 2014). Low Agreeableness and low Conscientiousness would be related to alcohol use through processes in which expectancies and other related social-cognitive constructs (i.e., motives) play a minor role: low Agreeableness-related characteristics would be associated with more problematic alcohol use through a Deviance Proneness pathway (Finn et al., 2000; Mezquita et al., 2014), whereas low Conscientiousness, which relates closely to Rash Impulsiveness (Gullo et al., 2010; Ibáñez et al., 2010), would be involved in alcohol use because of inhibitory control deficits which, for instance, would lead to difficulties in drinking refusal (Gullo et al., 2010).

We found a total mediation effect of negative AEs on alcohol problems and of positive AEs on alcohol use for Neuroticism, which is in line with findings reported in adults (Read and O’Connor, 2006). Thus high Neuroticism individuals tend to pay more attention to the negative outcomes of alcohol effects, as expected, but also to *all* positive AEs. Neuroticism includes the trait impulsivity, and it has been shown that impulsivity facets of Positive Urgency predict the development of positive AEs (Settles et al., 2010). However, we found that positive expectancies were associated with *all* Neuroticism facets, and not only with the impulsivity one (see Supplementary Table S4). Alternatively, it has been suggested that positive AEs may also include elements of negative reinforcement outcomes (Wardell et al., 2012). Therefore, high Neuroticism individuals would be more sensitive to the reduced negative affect (tension reduction AEs) in social and sexual contexts (Sexual and Social positive AEs), which could increase the positive affect (Fun AEs) associated with alcohol drinking.

Finally, we also found slight, but significant, moderation effects of Extraversion on the relationship between AEs and alcohol problems: extravert adolescents are more likely to be led to alcohol problems than introverts when a large number of positive or negative alcohol-related outcomes are experienced and anticipated. We propose two possible mechanisms underlying these interactions: vulnerability and exacerbation. Vulnerability suggests that the conjunction of the two risk variables would increase multiplicatively the probability for developing alcohol problems. Among Spanish teenagers, the more frequent pattern of alcohol consumption is known as “botellón,” which consists of frequent episodes of binge drinking and drunkenness at weekends, and takes place in groups and in open areas (Llorens et al., 2011). Thus, those adolescents high in excitement seeking and gregariousness that expected intense alcohol effects would be more involved in heavy drinking practices (such as “botellón”) that, in turn, could led to a more harmful and problematic pattern of alcohol use. Otherwise, according to the exacerbation explanation, it could be considered that expectancies would be indicative of the real effects that an adolescent experiences whilst drinking alcohol, and these intense effects would exacerbate the problems associated with alcohol use. As Extraversion is related to alcohol use, extraverted adolescents that experience high levels of alcohol effects would experience more alcohol problems. We think that these two speculative explanations could be applied differentially for the interaction of positive and negative expectancies, i.e., the vulnerability explanation could fit better for the Extraversion-Positive Expectancies interaction, whereas the exacerbation explanation could fit better for the Extraversion-Negative Expectancies interaction. In any case, the present findings highlight the synergistic effect between AEs and personality risk factors on alcohol problems in adolescence, although further research is needed in order to replicate the interaction effects and test possible underlying mechanisms.

Overall, and from a developmental perspective, adolescents and adults do not seem to present large qualitative differences in the mediational role of expectancies between FFM personality characteristics and alcohol outcomes. Thus Neuroticism and Extraversion (and related traits such as excitement and sensation seeking; Gullo et al., 2010) are associated with positive AEs, which in turn, are related to alcohol use in both adolescents (present study; Pilatti et al., 2012) and adults (Read and O’Connor, 2006; Gullo et al., 2010; Mezquita et al., 2015). In addition, Neuroticism would be associated with a more problematic alcohol use through negative expectancies in both developmental stages (present study; Read and O’Connor, 2006; Mezquita et al., 2015).

Regarding moderation effects, expectancies seem to present a small, albeit significant, interaction with personality traits on alcohol outcomes in both adolescents (present study) and adults (McCarthy et al., 2001; Cyders et al., 2007; Carlson and Johnson, 2012; Mezquita et al., 2015), although the personality domains differed for each developmental stage. Thus we describe in adolescence that Extraversion interacts with both positive and negative expectancies in predicting alcohol problems. In adulthood, positive AEs moderate the association between alcohol use and low Conscientiousness (Mezquita et al., 2015) and impulsivity/disinhibition (Carlson and Johnson, 2012). For alcohol problems, it has been reported interaction effects between positive expectancies and: (a) Neurotic Extraversion (McCarthy et al., 2001); (b) Neuroticism and low Agreeableness (Mezquita et al., 2015); and (c) Positive and Negative Urgency (Cyders et al., 2007). It is worth noting that both urgency facets are asssociated with Neuroticism (Cyders and Smith, 2008). The very few studies that have also examined the moderator role of negative expectancies have found that they tend to enhance the association between alcohol problems and Positive Urgency (Cyders et al., 2007) and low Agreeableness (Mezquita et al., 2015). Overall, in adolescence, the more important personality domain for problematic alcohol use when having high expectancies would be Extraversion; whereas in adulthood, it would be low Conscientiousness-related traits for alcohol use, and low Agreeableness and Neuroticism-related facets for alcohol problems.

This work has several limitations. The fact that our design is cross-sectional does not allow causal relationships to be established. Therefore, longitudinal cross-lagged studies can help determine the etiological links among personality traits, AEs, and alcohol use and misuse. Furthermore, the inclusion of other variables, such as motives to consume alcohol, parental environment, and rearing styles, peers influence, or psychopathological symptoms such as anxiety, depression or ADHD, among others, should provide a more comprehensive understanding of different drinking patterns.

Despite these limitations, the present study highlights the relevance of all FFM personality domains, and both positive and negative AEs, in adolescent alcohol use and misuse, and illustrates the complex interplay of these factors. Thus it emphasizes the benefit of using broad personality domains, of incorporating both positive and negative AEs, and of assessing different patterns of alcohol use to comprehensively explore adolescent drinking behavior. A better understanding of adolescent alcohol use determinants and outcomes may help improve prevention and treatment programs in earlier alcohol use development stages.

## Author Contributions

MI and GO designed the study. LC, LM, JM-H, and HV collected the data. LC and LM performed the statistical analyses. LC and MI wrote the first manuscript draft. All the authors contributed to and approved the final manuscript.

## Conflict of Interest Statement

The authors declare that the research was conducted in the absence of any commercial or financial relationships that could be construed as a potential conflict of interest.
