# Supplementary material for: Alcohol Expectancies Mediate and Moderate the Associations between Big Five Personality Traits and Adolescent Alcohol Consumption and Alcohol-Related Problems
Source: Front Psychol. 2015 Nov 26;6:1838. doi: 10.3389/fpsyg.2015.01838 (PMC4659872; doi:10.3389/fpsyg.2015.01838)
Supplement: Supplementary file 1 [file Table_1.DOCX]

Supplementary Material

Alcohol expectancies mediates and moderates the association between personality and adolescent drinking

Ibáñez, M.I., Camacho, L., Mezquita, L.*, Villa, H., Moya, J., Ortet, G.

*** Correspondence:** Corresponding Author: lmezquit@uji.es

# Supplementary Tables

**Supplementary Table 1. Means and standard deviations for the whole sample, and for males *vs.* females, and abstainers *vs.* drinkers, separately, *t* values and Cohen’s *d* associated with gender and drinking status.**

|  | Scales | α | Total sample  (*N* = 361) | Males  (*N* = 149) | Females  (*N* = 212) | *t-Test* | *d* | Abstainers  (*N* = 159) | Drinkers  (*N* = 202) | *t-Test* | *d* |
| --- | --- | --- | --- | --- | --- | --- | --- | --- | --- | --- | --- |
|  | Neuroticism | .86 | 60.92 (13.99) | 55.57 (13.77) | 64.68 (12.90) | -6.42** | .68 | 59.49 (14.33) | 62.04 (13.64) | -1.72 | .18 |
|  | Extraversion | .83 | 91.28 (12.37) | 90.05 (12.81) | 92.14 (12.01) | -1.59 | .17 | 88.59 (12.32) | 93.39 (12.02) | -3.72*** | .39 |
|  | Openness | .80 | 67.45 (12.09) | 64.01 (12.22) | 69.87 (11.41) | -4.66*** | .50 | 68.29 (12.16) | 66.79 (12.01) | 1.17 | .12 |
|  | Agreeableness | .78 | 74.47 (10.60) | 71.43 (10.92) | 76.61 (9.84) | -4.70*** | .50 | 74.62 (10.97) | 74.35 (10.32) | .24 | .03 |
|  | Conscientiousness | .90 | 84.31 (14.95) | 84.20 (16.14) | 84.38 (14.09) | -.12 | .01 | 86.89 (13.95) | 82.27 (15.43) | 2.95** | .32 |
|  | +S | .90 | 12.85 (7.14) | 12.13 (6.94) | 13.36 (7.25) | -.62 | .17 | 9.81 (6.97) | 15.24 (6.32) | -7.65*** | .82 |
|  | +F | .91 | 14.66 (7.41) | 13.95 (7.89) | 15.17 (7.03) | -1.51 | .16 | 10.70 (7.40) | 17.78 (5.76) | -9.91*** | 1.07 |
|  | +Sex | .92 | 6.29 (5.16) | 7.50 (5.25) | 5.43 (4.93) | 3.81*** | .41 | 4.32 (4.46) | 7.83 (5.15) | -6.93*** | .73 |
|  | +T | .78 | 6.01 (3.67) | 5.81 (3.85) | 6.16 (3.53) | -.88 | .09 | 4.72 (3.63) | 7.02 (3.36) | -6.15*** | .66 |
|  | -S | .82 | 2.70 (3.01) | 3.53 (3.34) | 2.12 (2.62) | 4.30*** | .47 | 2.70 (3.32) | 2.71 (2.76) | -0.31 | .00 |
|  | -E | .74 | 3.47 (3.00) | 3.53 (3.19) | 3.43 (2.87) | .30 | .03 | 3.56 (3.35) | 3.40 (2.70) | .47 | .05 |
|  | -P | .80 | 6.99 (4.68) | 6.67 (4.71) | 7.21 (4.66) | -1.08 | .12 | 6.82 (5.44) | 7.12 (3.99) | -0.57 | .06 |
|  | -C | .87 | 9.98 (5.97) | 9.96 (6.35) | 9.99 (5.71) | -.05 | .01 | 8.66 (6.42) | 11.00 (5.40) | -3.64*** | .39 |
|  | Positive AEs | .95 | 39.81 (20.46) | 39.38 (21.50) | 40.11 (19.75) | -.33 | .04 | 29.57 (19.87) | 47.88 (17.07) | -9.24*** | .99 |
|  | Negative AEs | .92 | 23.14 (13.90) | 23.69 (14.79) | 22.76 (13.27) | .63 | .07 | 21.76 (16.13) | 24.23 (11.79) | -1.61 | .17 |
|  | Weekday SDUs | - | .58 (2.13) | .99 (2.72) | .30 (1.55) | 2.79** | .31 | 0 (0) | 1.04 (2.77) | -5.33*** | .53 |
|  | Weekend SDUs | - | 4.88 (6.64) | 5.82 (7.71) | 4.21 (5.70) | 2.15* | .24 | 0 (0) | 8.71 (6.74) | -18.38*** | 1.83 |
|  | Alcohol Problems | .72 | 1.25 (2.50) | 1.28 (2.92) | 1.22 (2.17) | .23 | .02 | 0.39 (1.41) | 1.22 (2.17) | -6.51*** | .45 |

+S = Positive Social, +F = Fun, +T = Tension Reduction, -S = Negative Social, -E = Negative Emotional, -P = Physical Effects and –C = Cognitive Performance; AEs = Alcohol Expectancies; SDUs = Standard Drink Units. Rank anchors: JS NEO scales 0-120, Positive AEs 0-95, Negative AEs 0-75, AP 0-28. Cohen´s *d* (in absolute values) of .20, .50 and .80 indicate small, moderate and large size effects, respectively (Cohen, 1992).

**p*< .05; ***p*< .01; ****p*< .001.

**
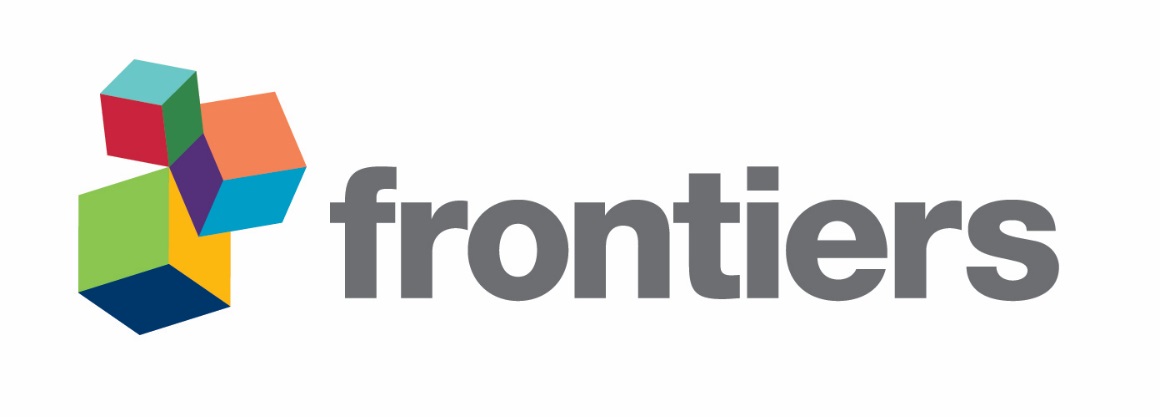
**
