# Supplementary material for: Alcohol Expectancies Mediate and Moderate the Associations between Big Five Personality Traits and Adolescent Alcohol Consumption and Alcohol-Related Problems
Source: Front Psychol. 2015 Nov 26;6:1838. doi: 10.3389/fpsyg.2015.01838 (PMC4659872; doi:10.3389/fpsyg.2015.01838)
Supplement: Supplementary file 2 [file Table_2.DOCX]

Supplementary Material

Alcohol expectancies mediates and moderates the association between personality and adolescent drinking

Ibáñez, M.I., Camacho, L., Mezquita, L.*, Villa, H., Moya, J., Ortet, G.

*** Correspondence:** Corresponding Author: lmezquit@uji.es

# Supplementary Tables

**Supplementary Table 2. Hierarchical regression analysis with Positive and Negative Alcohol Expectancies (AEs) as the dependent variables; and Personality, Drinking Status, and their interaction as predictors (*N* = 361).**

|  |  | Positive AEs | Negative AEs |
| --- | --- | --- | --- |

|  |  | ∆R^2^ | β | ∆R^2^ | β |
| --- | --- | --- | --- | --- | --- |
| Step 1 | Gender (0 = male; 1 = female) | .000 | .018 | .002 | -.032 |
|  | Age |  | -.005 |  | -.023 |
| Step 2 | Neuroticism (N) | .099*** | .206** | .070*** | .154* |
|  | Extraversion (E) |  | .215*** |  | .003 |
|  | Openness (O) |  | .049 |  | .049 |
|  | Agreeableness (A) |  | -.089 |  | -.136* |
|  | Conscientiousness (C) |  | -.085 |  | -.090 |
| Step 3 | Drinking status  (0 = abstainers; 1 = drinkers) | .154*** | .414*** | .004 | .069 |
| Step 4 | Drinking status x N | .006 | .011 | .010 | -.060 |
|  | Drinking status x E |  | .048 |  | .068 |
|  | Drinking status x O |  | -.079 |  | -.097 |
|  | Drinking status x A |  | .079 |  | .109 |
|  | Drinking status x C |  | .069 |  | .005 |

**p* < .05; ***p* < .01; ****p* < .001.

**
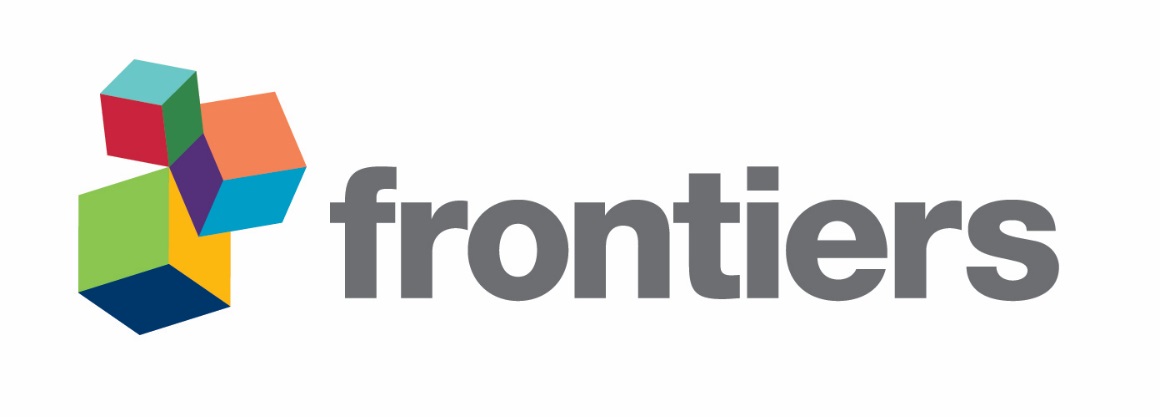
**
