# Supplementary material for: Alcohol Expectancies Mediate and Moderate the Associations between Big Five Personality Traits and Adolescent Alcohol Consumption and Alcohol-Related Problems
Source: Front Psychol. 2015 Nov 26;6:1838. doi: 10.3389/fpsyg.2015.01838 (PMC4659872; doi:10.3389/fpsyg.2015.01838)
Supplement: Supplementary file 3 [file Table_3.DOCX]

Supplementary Material

Alcohol expectancies mediates and moderates the association between personality and adolescent drinking

Ibáñez, M.I., Camacho, L., Mezquita, L.*, Villa, H., Moya, J., Ortet, G.

*** Correspondence:** Corresponding Author: lmezquit@uji.es

# Supplementary Tables

**Supplementary Table 3.**  **Hierarchical regression analysis with Alcohol Problems as the dependent variable; and Personality, Alcohol Expectancies (AEs) and their interaction as predictors (*N* = 361).**

|  |  | Alcohol Problems | | | | | |
| --- | --- | --- | --- | --- | --- | --- | --- |
|  |  | ∆R^2^ | | | | β | |
| Step 1 | Gender (0 = male; 1 = female) | .000 | | | | -.011 | |
|  | Age |  | | | | -.009 | |
| Step 2 | Neuroticism | .075*** | | | | .027 | |
|  | Extraversion |  | | | | .196*** | |
|  | Openness |  | | | | -.038 | |
|  | Agreeableness |  | | | | -.142* | |
|  | Conscientiousness |  | | | | -.133* | |
|  |  | | ∆R^2^ | β | ∆R^2^ | | β |
| Step 3 | Positive AEs  Negative AEs | | .054*** | .244*** | .026** | | .167** |
| Step 4 | AEs^1^ x Neuroticism | | .023 | .026 | .045** | | .031 |
|  | AEs^1^ x Extraversion | |  | .141** |  | | .212*** |
|  | AEs^1^ x Openness | |  | -.055 |  | | -.081 |
|  | AEs^1^ x Agreeableness | |  | -.078 |  | | .012 |
|  | AEs^1^ x Conscientiousness | |  | -.018 |  | | -.043 |

Note. ^1^Interactions between Alcohol Expectancies and personality have been examined separately for positive and negative AEs.

**p* < .05; ***p* < .01; ****p* < .001.

**
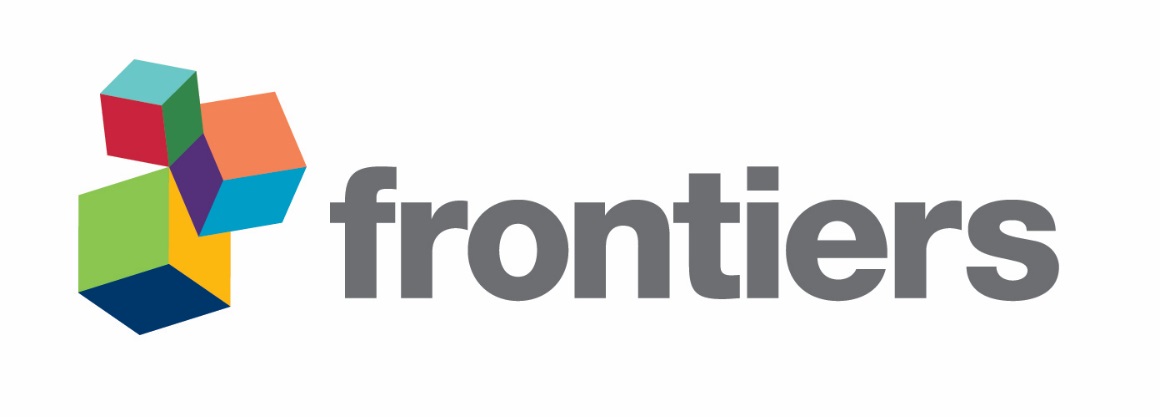
**
