# Supplementary material for: Alcohol Expectancies Mediate and Moderate the Associations between Big Five Personality Traits and Adolescent Alcohol Consumption and Alcohol-Related Problems
Source: Front Psychol. 2015 Nov 26;6:1838. doi: 10.3389/fpsyg.2015.01838 (PMC4659872; doi:10.3389/fpsyg.2015.01838)
Supplement: Supplementary file 4 [file Table_4.DOCX]

Supplementary Material

Alcohol expectancies mediate and moderate the association between personality and adolescent drinking

Ibáñez, M.I., Camacho, L., Mezquita, L.*, Villa, H., Moya, J., Ortet, G.

*** Correspondence:** Corresponding Author: lmezquit@uji.es

# Supplementary Tables

**Supplementary Table 4. Correlations among FFM personality facets and expectancies and alcohol outcomes**

|  | N1 | N2 | N3 | N4 | N5 | N6 | E1 | E2 | E3 | E4 | E5 | E6 |
| --- | --- | --- | --- | --- | --- | --- | --- | --- | --- | --- | --- | --- |
| Positive AE | .12* | .14** | .16** | .16** | .15** | .15** | .07 | .06 | .01 | -.07 | .18*** | .09 |
| Negative AE | .10 | .20*** | .13* | .15** | .11* | -.06 | -.09 | -.09 | -.10 | .09 | .04 | -.07 |
| Weekday SDUs | -.03 | -.01 | -.02 | .02 | -.01 | -.11* | -.11* | .02 | .07 | .01 | .08 | -.06 |
| Weekend SDUs | .00 | .00 | .04 | .04 | .10 | .00 | .07 | .11* | .06 | -.13* | .16** | .08 |
| Binge drinking^+^ | .07 | .07 | .12* | .08 | .19*** | .09 | .14** | .18*** | .07 | -.11* | .25*** | .09 |
| Alcohol Problems (AP) | .05 | .06 | .06 | .01 | .12* | .03 | .02 | .15** | .15** | -.06 | .21*** | .02 |

*Note.* AE = Alcohol Expectancies; SDUs = Standard Drink Units; Binge drinking^+^ = Item 3 from AUDIT (“How often do you have six drinks or more on one occasion?”); N1 = Anxiety; N2 = Angry Hostility; N3 = Depression; N4 = Self-Consciousness; N5 = Impulsiveness; N6 = Vulnerability; E1 = Warmth; E2 = Gregariousness; E3 = Assertiveness; E4 = Activity; E5 = Excitement Seeking; E6 = Positive Emotions

**p* < .05; ***p* < .01; ****p* < .001.

**Supplementary Table 4 (cont.)**

|  | A1 | A2 | A3 | A4 | A5 | A6 | C1 | C2 | C3 | C4 | C5 | C6 |
| --- | --- | --- | --- | --- | --- | --- | --- | --- | --- | --- | --- | --- |
| Positive AE | .01 | -.18** | -.02 | -.10 | -.07 | -.01 | -.09 | -.20*** | -.13* | -.08 | -.06 | -.14** |
| Negative AE | -.10 | -.19*** | .01 | -.12* | -.13* | .00 | -.12* | -.22*** | -.17*** | -.09 | -.12** | -.09 |
| Weekday SDUs | -.08 | -.10 | -.01 | -.12* | -.13* | -.07 | -.04 | .03 | -.03 | .00 | -.01 | -.04 |
| Weekend SDUs | -.01 | -.09 | .05 | -.09 | .03 | .01 | -.09 | -.06 | -.07 | -.04 | -.08 | -.21*** |
| Binge drinking^+^ | .03 | -.11* | .06 | -.12* | .04 | -.00 | -.10* | -.13* | -.09 | -.08 | -.13* | -.16** |
| Alcohol Problems (AP) | -.10* | -.13* | .05 | -.13* | -.05 | -.11* | -.11* | -.11* | -.19*** | -.15** | -.10 | -.12* |

*Note.* AE = Alcohol Expectancies; SDUs = Standard Drink Units; Binge drinking^+^ = Item 3 from AUDIT (“How often do you have six drinks or more on one occasion?”); A1 = Trust; A2 = Straightforwardness; A3 = Altruism; A4 = Compliance; A5 = Modesty; A6 = Tender-Mindedness; C1= Competence; C2 = Order; C3 = Dutifulness; C4 = Achievement Striving; C5 = Self-Discipline; C6 = Deliberation

**p* < .05; ***p* < .01; ****p* < .001.

**Supplementary Table 4 (cont.)**

|  | O1 | O2 | O3 | O4 | O5 | O6 |
| --- | --- | --- | --- | --- | --- | --- |
| Positive AE | .02 | .00 | .06 | .07 | .04 | -.06 |
| Negative AE | .00 | -.03 | -.03 | .04 | .06 | -.02 |
| Weekday SDUs | -.07 | -.01 | -.03 | -.01 | -.01 | .10 |
| Weekend SDUs | -.05 | -.14** | -.01 | -.04 | -.05 | .14** |
| Binge drinking^+^ | -.02 | -.06 | .07 | .03 | -.05 | .03 |
| Alcohol Problems (AP) | -.06 | -.05 | -.01 | -.02 | -.09 | .07 |

*Note.* AE = Alcohol Expectancies; SDUs = Standard Drink Units; Binge drinking^+^ = Item 3 from AUDIT (“How often do you have six drinks or more on one occasion?”); O1 = Fantasy; O2 = Aesthetics; O3 = Feelings; O4 =Actions; O5 = Ideas; O6 = Values

**p* < .05; ***p* < .01; ****p* < .001.

**
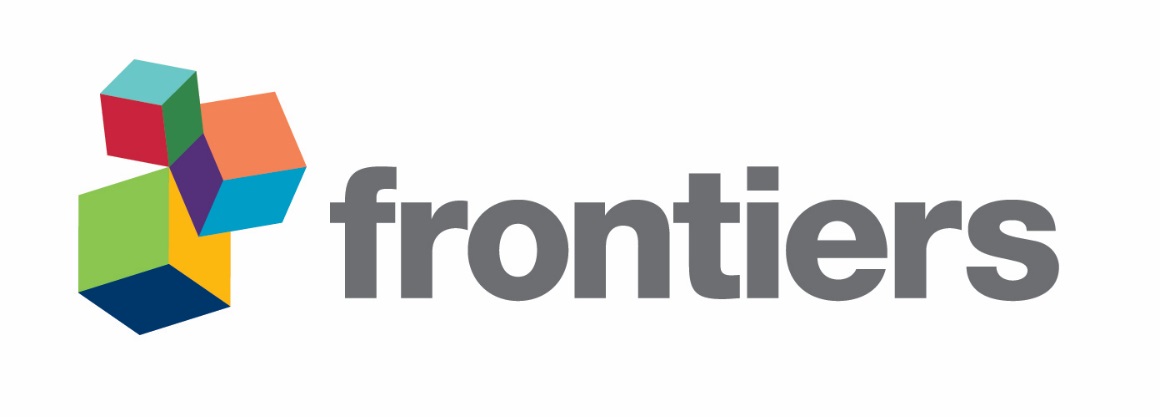
**
