# Supplementary material for: Alcohol Expectancies Mediate and Moderate the Associations between Big Five Personality Traits and Adolescent Alcohol Consumption and Alcohol-Related Problems
Source: Front Psychol. 2015 Nov 26;6:1838. doi: 10.3389/fpsyg.2015.01838 (PMC4659872; doi:10.3389/fpsyg.2015.01838)
Supplement: Supplementary file 5 [file Table_5.DOCX]

Supplementary Material

Alcohol expectancies mediates and moderates the association between personality and adolescent drinking

Ibáñez, M.I., Camacho, L., Mezquita, L.*, Villa, H., Moya, J., Ortet, G.

*** Correspondence:** Corresponding Author: lmezquit@uji.es

# Supplementary Tables

**Supplementary Table 5. Partial correlations controlled by gender.**

|  | +AE | +S | +F | +Sex | +T | -AE | -S | -E | -P | -C | Weekday SDUs | Weekend SDUs | Binge Drinking | AP |
| --- | --- | --- | --- | --- | --- | --- | --- | --- | --- | --- | --- | --- | --- | --- |
| Neuroticism | .21*** | .21*** | .17** | .16** | .21*** | .21*** | .04 | .22*** | .18** | .22*** | .02 | .09 | .14** | .08 |
| Extraversion | .16** | .10 | .23*** | .12* | .07 | -.06 | .01 | -.11* | -.08 | -.02 | .02 | .18** | .23*** | .14** |
| Openness | .03 | .04 | .02 | .01 | .04 | -.01 | -.06 | .06 | -.04 | .01 | -.00 | -.08 | -.02 | -.07 |
| Agreeableness | -.10 | -.03 | -.06 | -.17** | -.12* | -.18** | -.22*** | -.09 | -.15** | -.15** | -.12* | .01 | -.05 | -.15** |
| Conscientiousness | -.16** | -.12* | -.16** | -.11* | -.19*** | -.18** | -.09 | -.07 | -.15** | -.23*** | -.01 | -.13* | -.16** | -.17** |
| Weekday SDUs | .11* | .07 | .10 | .13* | .13* | .03 | .09 | -.03 | -.01 | .05 | 1.00 | .46*** | .26*** | .36*** |
| Weekend SDUs | .38*** | .30*** | .40*** | .32*** | .30*** | .09 | .05 | -.05 | .04 | .17** | .46*** | 1.00 | .58*** | .40*** |
| Binge drinking^+^ | .41*** | .32*** | .45*** | .28*** | .35*** | .19*** | .00 | -.03 | .11* | .30*** | .26*** | .58*** | 1.00 | .51*** |
| Alcohol Problems (AP) | .29*** | .22*** | .30*** | .24*** | .27*** | .20*** | .17** | .05 | .12* | .26*** | .36*** | .40*** | .51*** | 1.00 |

*Note.* +AE = Positive Alcohol Expectancies; +S = Positive Social, +F = Fun, +T = Tension Reduction, -AE = Negative Alcohol Expectancies; -S = Negative Social, -E = Negative Emotional, -P = Physical Effects and –C = Cognitive Performance; SDUs = Standard Drink Units; Binge drinking^+^ = Item 3 from AUDIT (“How often do you have six drinks or more on one occasion?”)

**p*< .05; ***p*< .01; ****p*< .001.

**
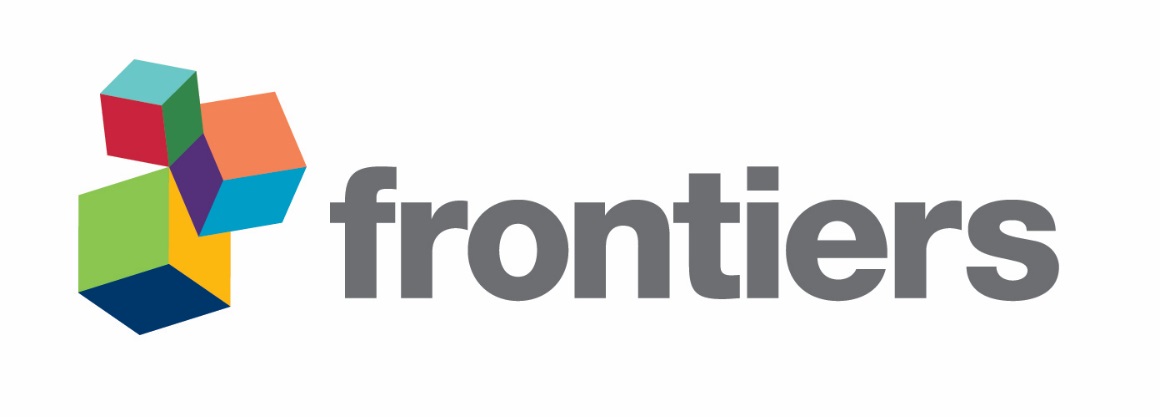
**
